# Re-expansion and Stabilization of Vertebra Plana Fractures Using Bilateral SpineJack® Implants

**DOI:** 10.7759/cureus.13839

**Published:** 2021-03-11

**Authors:** David M Joyce, Michelle Granville, Robert E Jacobson

**Affiliations:** 1 Pain Management, Larkin Community Hospital, Miami, USA; 2 Neurosurgery, University of Miami Hospital, Miami, USA; 3 Neurological Surgery, University of Miami Hospital, Miami, USA

**Keywords:** vertebra plana, osteoporotic vertebral fractures, spinejack, vertebral clefts

## Abstract

The surgical treatment of osteoporotic vertebral fractures with greater than 70% collapse, known as "Vertebra Plana (VP)" has been controversial. Originally VP was a considered a contraindication to vertebroplasty or kyphoplasty because of presumed difficulty of entering the collapsed vertebra as well as obtaining significant re-expansion or correct associated sagittal kyphosis. In some cases, multilevel pedicle screw fixation with or without attempts to correct the collapse is still performed to correct the kyphosis or prevent progression. With experience it was clear that the pedicle could be accessed and VP could be treated without added risk of epidural leak of cement or epidural extravasation. Now, with the introduction of newer third-generation intraspinal expansion devices that are larger and need to be placed bilaterally, their use in cases of VP was again an issue since VP cases were excluded from the original multicenter studies used for worldwide approval. This report reviews six cases of VP treated with bilateral SpineJack® implants (Stryker Corp, Kalamazoo, Michigan, USA) demonstrating it is not only feasible to place these larger size implants but achieve significant reconstitution of vertebral height as well as correction of the kyphotic deformity.

## Introduction

The surgical treatment of osteoporotic vertebral fractures, when greater than 70% collapse, commonly known as “Vertebra Plana” (VP), has been controversial. Originally it was felt that such severe vertebral collapse could not be treated with either vertebroplasty or balloon kyphoplasty because of the difficulty of entering the collapsed vertebra, reducing posterior vertebral endplate fragments, or obtaining and maintaining significant re-expansion, as well as difficulty in correction of frequently associated sagittal kyphosis [1]. It was recommended to perform multilevel pedicle screw fixation but this was problematic in already osteoporotic elderly patients, where pedicle screws in adjacent osteoporotic vertebrae would loosen or not hold, as well as the elderly patients having significant medical co-morbidities that precluded a major spinal surgical procedure. Often fixation was done without correction of the collapsed vertebrae or correction of the kyphosis, which led to further fracture progression [2,3]. Recently, short segment pedicle fixation combined with kyphoplasty or vertebral corpectomy and vertebral replacement was suggested to improve the kyphosis [4]. More recent studies demonstrated that the pedicle and collapsed vertebrae could be accessed using either a lateral or transpedicular approach for simple vertebroplasty of kyphoplasty; however, it was difficult to restore vertebral height or correct kyphosis [5,6]. Over the last 10 years, the evolution of third-generation intravertebral implants demonstrates that implants often can provide significant height correction beyond what occurs with balloon kyphoplasty and that this correction can be maintained over one or more years. Follow-up studies have also shown that these permanently expanded implants are associated with statistically less adjacent level fractures [7,8]. VP cases were specifically excluded from the early SpineJack® (SJ) studies (Stryker Corp, Kalamazoo, Michigan, USA) [7]. This report reviews six cases of VP treated with bilateral SJ implants. These cases show it is feasible to place these larger implants bilaterally in VP cases and that these implants provide significant lasting vertebral height reconstitution and correction of the kyphotic deformity.

Radiologic evaluation of the fractured vertebrae in VP is important, and the degree of actual collapse seen on plain radiographs can be deceptive [9,10]. In the patient with a VP fracture, besides magnetic resonance imaging (MRI) and computerized tomography (CT) scans, the additional evaluation of both static as well as functional flexion/extension films provides added information and is a key step of the surgical planning of VP fractures [10-12]. Radiologic studies evaluate if there is sagittal angulation or kyphosis, coronal collapse, measurement of the size of any posterior displaced endplate fragments, presence of fracture lines or comminuted endplates extending along the adjacent disk space, or fracture lines extending to the posterior vertebral wall and especially the presence of vertebral clefts [13]. Intravertebral clefts within the fractured or VP vertebrae, either seen on CT or MRI scan, are a clear sign of vertebral motion and instability. Flexion/extension motion films or placing the patient in extension before or during surgery can show movement and enlargement of the cleft and actual change in size of the “collapsed” vertebrae in VP, indicating that the fracture is more likely to be able to be re-expanded to some degree [13,14]. Becker et al. reported in 2008 a comparative study of the importance of a vertebral cleft using balloon kyphoplasty in 13 cases of VP. He examined the change in angulation by comparing the mid and anterior vertebral body height after the procedure in cases with and without the cleft showing cases with clefts expanded more readily [13]. Generally, long-term follow-up of balloon kyphoplasty cases show gradual loss of this height correction, ranging anywhere as short as one month to over a year after the procedure. This led to the development of various “third-generation” implants for vertebral fractures that rely on placement of an implantable permanent support within the vertebral body to both get initial height correction and more importantly to maintain that correction [7]. In 2019, the Study to Compare Safety and Effectiveness of Two Vertebral Compression Fracture Reduction Techniques (SAKOS study) compared SJ to balloon kyphoplasty. The SAKOS as well as several other subsequent studies confirmed these expandable implants not only created better initial height correction, especially in the anterior and middle part of the vertebrae, but its superiority was maintained in long-term follow-up up to one year with an additional finding of significantly less adjacent level fractures. The SAKOS study, however, specifically excluded VP patients that we address in this report [7,8].

Our experience in these VP cases provides an insight into both the radiologic parameters and the specific surgical considerations in placing these larger diameter implants in severely collapsed VP patients. There are also some additional technical steps in doing these VP cases, and these specific steps correlate with the anatomic and pathologic changes that occur with severe vertebral body collapse. The implants, before being expanded, are inserted in one of three sizes that are predetermined by measurements utilizing CT or MRI scans. The closed implants are inserted through specially designed cannulas that are 4.2, 5.0, and 5.8 mm in diameter, which are sequentially larger than the normal “jamshidi” style cannula used for vertebral access in vertebroplasty or balloon kyphoplasty, which normally range in external diameter from 1.8 mm (13 gauge) to 4.2 mm (8 gauge).

## Materials and methods

A retrospective chart review was made of all patients that had undergone balloon kyphoplasty, vertebroplasty, or SJ implants over a 24-month period of time. All cases of VP determined by CT/MRI whether they had vertebroplasty, balloon kyphoplasty, or SpineJack procedure were reviewed, and the SJ implant cases were separated. The history and interval from injury to initial and any subsequent radiologic studies were recorded. Bone mineral density (BMD), treatment for osteoporosis, age, level, and use of any medication such as cortico-steroids was recorded. The level and size of device implanted were noted. A pre-procedure visual analog scale (VAS) score was obtained as along with initial post-procedure and at least a one-month follow-up score. Comparison of vertebral sagittal heights in anterior, middle, and posterior vertebra plus any angulation was made from pre- and post-procedure films. Any extravasation of cement into the disk or epidural space was noted.

Radiologic studies

When possible, patients had plain x-rays, CT, and MRI scans. Depending on timing from initial onset/injury and first neurosurgical evaluation, repeat scans were performed and flexion/extension films were obtained to evaluate if there was any movement or further progression of collapse of the fracture. If there was history of previous fractures, a bone scan was performed, and the most recent BMD was checked.

Surgical technique

All patients were medically evaluated, and when cleared, the patients had the procedure performed in an ambulatory outpatient surgery setting, with local anesthesia through mild sedation as needed. All patients were discharged home with office follow-up. No patient had immediate or delayed hospital admission after the procedure. Follow-up was performed in office at one and four to six weeks after the procedure, and both plain x-rays and usually a CT scan were performed for follow-up measurements. A preoperative CT scan was used to make measurements of pedicle diameter and vertebral depth for sizing of the implants. The basic surgical technique is as described in the literature using a completely percutaneous, fluoroscopically guided approach used in both vertebroplasty and kyphoplasty. The sequential steps are placement of the initial vertebral access cannula, “k wire,” second longer access cannula with reamer and template, then insertion and expansion of the bilateral implants as alternate steps, and then filling the expanded implant with polymethyl-methacrylate (PMMA) cement. However, one additional step and modification was added in the VP cases. After the initial standard length cannula was placed, a malleable curved curette that was long enough to fit past the access cannula was used to dissect along the cleft especially toward the midline on both sides. This allowed opening of the vertebral cleft assisting the insertion and expansion of the closed SJ device. It needed to be done at this stage since once the secondary larger cannula was used with the reamer, the curette was too short to be able to sweep into the vertebral cleft. After this step, the reamer, template, and SJ implant were placed so open to the cleft more toward the midline, thereby enabling better expansion.

## Results

A chart review revealed that over the 24 months, a total of 37 patients had SJ devices implanted for fractures at one or more levels. An additional 27 patients had either vertebroplasty or balloon kyphoplasty. Of these 64 patients, 14 had VP. Six patients underwent vertebral augmentation with bilateral SJ implants for single-level VP fractures, while the remaining eight had either vertebroplasty or balloon kyphoplasty. All VP fractures treated with SJ were in the thoraco-lumbar junction; two patients had fractures at T12, three at L1, and one at L2. Two of the six patients had clear vertebral clefts with vertebral body edema on CT and MRI scan, while another two had suggestion of clefts only on one examination but not on both CT and MRI. Posterior superior endplate fragments were seen in three patients and varied from 2 to 6 mm, but no patient had sagittal canal compromise greater than 50%. Preoperative angulation varied from zero to 37 degrees, but four of six had only between seven to 10 degrees of angulation. Post procedure, angulation in those four patients improved from zero to four degrees. The average height increase after implant compared to pre-procedure sagittal films was 14 mm in the mid-vertebrae and 12 mm in the anterior vertebrae, consistent with the vertical heights of the sizes of the fully expanded implants used. Pain measured by VAS decreased 60% from an average of 8.1 before the surgery to 3.3 within 48 hours after the procedure. At one-month follow-up, pain further decreased to a VAS of 2.4 (Table 1).

**Table 1:** Results RX TX, Pharmacologic treatment for osteoporosis; BMD, bone mineral density; pre-VAS, visual analog score at initial office visit; post-VAS, visual analog score at post-operative visit; f, female; m, male; L, lumbar; T, thoracic; d, days; mm, millimeter.

Age/gender	Level	Time to surgery	Procedure	RX TX	BMD	Pre-VAS	Post-VAS	Vertebral cleft	Posterior fragment	Angulation
80 f	T12	26 d	Bilateral T12 insertion 4.2 mm SpineJack® devices	No	None	8	3	Yes	No	37 degrees
96 f	T12	7 d	Bilateral T12 insertion 4.2 mm SpineJack® devices	Yes	AP spine - 0.0	6	0	No	No	None
91 f	L1	8 d	Bilateral L1 insertion 4.2 mm SpineJack® devices	No	AP spine - 2.3	9	3	Yes	No	10 degrees
80 f	L2	5 d	Bilateral L2 insertion 5.0 mm SpineJack® devices	Yes	None	10	6	No	5 mm	8 degrees
83 f	L1	19 d	Bilateral L1 insertion 5.0 mm SpineJack® devices	Yes	None	7	2	No	4.7 mm	7 degrees
73 m	L1	*192 d	Bilateral L1 insertion 5.0 mm SpineJack® devices	Yes	AP spine - 2.9	9	6	No	2.3 mm	8 degrees

## Discussion

**Figure 1:**
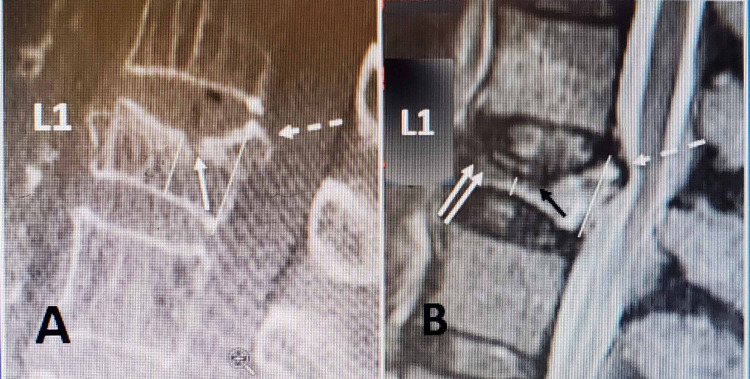
Patient with an acute L1 fracture seen on MRI and compared with bone detail seen on CT scan. CT demonstrates that fracture has progressed in less than four weeks to a vertebra plana. A: CT scan performed two weeks after fall of a L1 fracture showing fracture in middle and slightly posterior superior endplate (solid white arrow). There is a 3.5 mm posteriorly displaced superior endplate (dashed white arrow). The degree of collapse is approximately 50% indicated by thin solid white arrows posteriorly and in middle of the collapsed vertebra. B: MRI scan on same patient after being seen four weeks later compared to CT scan. The collapse has progressed to 80% (small solid white arrows), and the fracture of the superior endplate (double white arrows) has become more severe in the middle and anterior areas. The solid black arrow shows the progressive mid-vertebral collapse that occurred in the interval from CT to MRI scan. The posteriorly displaced superior endplate is now over 5 mm in size and extends more inferiorly compared to what was seen on CT scan (dashed white arrow) and is involving the middle of the posterior edge of vertebra wall abutting the spinal canal. CT, Computerized tomography; MRI, magnetic resonance imaging.

Accurate measurements and repeat imaging to evaluate if there has been any fracture progression are essential in surgical planning for SJ placement in VP cases. Final pre-procedure imaging after positioning in extension on the procedure table has been shown to cause some re-expansion of a severely collapsed vertebrae especially if there is an intravertebral cleft [10,11]. In making a pre-surgical evaluation of acute and subacute VP fractures, factors to be considered are the percentage of fracture collapse, the presence of an intravertebral cleft, the existence and size of any posterior displaced endplate fragment(s) into the canal, and the degree of kyphotic angulation of both the individual vertebrae and the spine. The utilization of multiple imaging modalities including plain x-rays, dynamic films, CT, and MRI provides different types of information that when combined give a more precise understanding of the various pathologic elements of each individual fracture [9-12]. Each imaging modality serves an important role in establishing if there has been any progression in the fracture from the time of initial patient presentation to completion of the implant procedure. It is important to compare sequential films, if available, or to consider re-evaluation if more than several weeks occur from the radiographs at the time of initial injury until the date of surgery since fractures can easily progress from partial to more severe collapse with the development of more severe kyphotic deformity in a short time frame affecting the planned procedure (Figure 1).

CT sagittal views are especially useful in following any progression of collapse and change in angulation, both of which alter the biomechanics of the spine and increase the propensity of adjacent level fractures, potentially necessitating a change in surgical strategy. CT can identify vertebral clefts that show up as hypodense “black” clefts within the vertebrae often with associated “vacuum” changes in the adjacent disc space. These are sometimes noted as "vacuum phenomenon" in radiologic reports and are seen both within the collapsed vertebrae and can be also identified within the adjacent disc space. Coronal views will show any asymmetric collapse that may not be as clear on plain x-rays [13,14]. Additionally, day of surgery fluoroscopic scout AP and lateral views provide a final verification that no further progression has taken place, and often with patient positioning in the prone position and some extension, there can be an actual expansion in the fracture [15].

MRI reveals the presence of edema within the fracture on T1- and T2-weighted scans. Additionally, in patients with progressive kyphosis, early edematous changes in the adjacent vertebra are closely correlated with the progression of kyphosis. The presence of a cleft is probably the most significant radiologic finding with vertebral fractures [6,13]. Of the six patients who underwent bilateral SJ placement, five presented with VP, and three of six patients were found to have vertebral clefts, illustrating the potential for further collapse even after VP has been initially identified. The presence of a cleft when seen on CT as a hypodense line or “vacuum change” and on MRI as a high-intensity signal on T2 and Short Tau Inversion Recovery (STIR) sequences is indicative of fluid with high water content, a sign of micro-motion within the fracture, osteonecrosis, and instability of the fracture [6,13]. For this reason, plain flexion/extension radiographs are important to detect fracture movement, especially if a vertebral cleft is initially identified [11,12,15]. In cases with clefts, movement of the fracture within the vertebrae with flexion and extension films can show the cleft enlarging, and this motion is reported to correlate with either a balloon or internal expanding device being especially effective in restoring vertebral height [13]. It is clear in the literature that with the existence of a vertebral cleft regardless of the approach used (vertebroplasty, kyphoplasty, or implants), it is critical at the time of the procedure to ensure cement fill within the cleft, or there is a much higher incidence of progressive collapse and eventual failure of the procedure can occur [6,12,13,16,17].

In cases planning to use a SJ device, CT scan provides more accurate measurements of the pedicle width in axial view, which is used for sizing of the closed implant diameter and determining overall depth within the fractured vertebral body [7,8,13]. CT also gives detailed information in any fracture lines through the endplates and posterior vertebral wall that may be related to increased risk of cement leakage either into the disk space or epidural space [14].

While VP has been considered a contraindication in the past for vertebral augmentation and especially for VP, we have shown it was possible to successfully place a vertebroplasty or kyphoplasty cannula as well as larger SJ cannulas in all VP without complications. Due to the limited potential space for a relatively larger implant, combined with considerations of pedicle size and location, precision in measurements is paramount to proper device placement and optimal outcome.

The SJ device is available in three sizes based on closed diameter, each with different load characteristics and fully expanded length and height. In vertebral fractures with severely collapsed vertebrae, not only is the diameter of the pedicle a factor but eventual full expansion height is important. The 4.2 SJ expands to 12.5 mm, the 5.0 SJ to 17 mm, and the 5.8 SJ to 20 mm; so there is a 4.5 mm difference allowing potentially greater height correction if the 5.0 mm implant can be placed rather than the 4.2 mm size. Of the six patients who underwent successfully bilateral SJ placement, all fractures were centered at the thoraco-lumbar junction from T11 to L2, where 4.2 and 5.0 mm implants were used. To determine device size and optimal placement, multiple measurements are made, preferably with CT. The final expansion size of the implant determines the overall vertebral expansion obtained with the implant; so our feeling is to try and use the largest implant based on pedicle size.

To determine implant size, axial and coronal pedicle diameter is measured to determine cannula size, potentially limiting the maximal size of the device. Additionally, both sagittal and axial views are correlated to determine the optimal implant angles and final size and depth of the implants. In the sagittal view, the length of the vertebral body is measured from a point just anterior to the pedicle to the anterior vertebral margin. Axial views allow assessment of optimal angle of convergence while estimating the angle for safe transpedicular approach. Ideal placement of the implants is positioning posteriorly just from the vertebral base of the pedicle and passing anteriorly just lateral of the midline so that both implants together form a "V" with the maximum support along a line from the pedicle proceeding bilaterally in the anterior part of the vertebral body [7,8,16]. This provides for best expansion and mechanical support especially in the maximum area of collapse in the mid and anterior vertebral body.

Additionally, CT also gives a very accurate image of any fracture lines extending through the posterior wall adjacent to the spinal canal and endplates as well as the ability to visualize posterior fragments and provide a clearer picture of the size and location of these bone fragments [9,10]. The precise “mm” displacement and percentage of canal compromise seen on CT or MRI are important, and unless it is greater than 50% it is rare to see cord or root compression even in the thoraco-lumbar area since this includes the smaller conus and cauda equina. It has been established that re-expansion of the vertebrae will re-align the fragment through the phenomenon of ligament-taxis both during pedicle fixation as well as vertebral expansion with kyphoplasty since the displaced fragment of the superior endplate is attached to the posterior longitudinal ligament (PLL) that “restrains” the fragment and approximates it closer back to the fractured vertebrae with re-expansion [4,11]. Axial views will show the side of displacement of the fragment, and coronal views show asymmetric collapse of the endplate since it is critical to access the more compressed side to avoid later failure of kyphoplasty or implant procedures [6,10].

**Figure 2:**
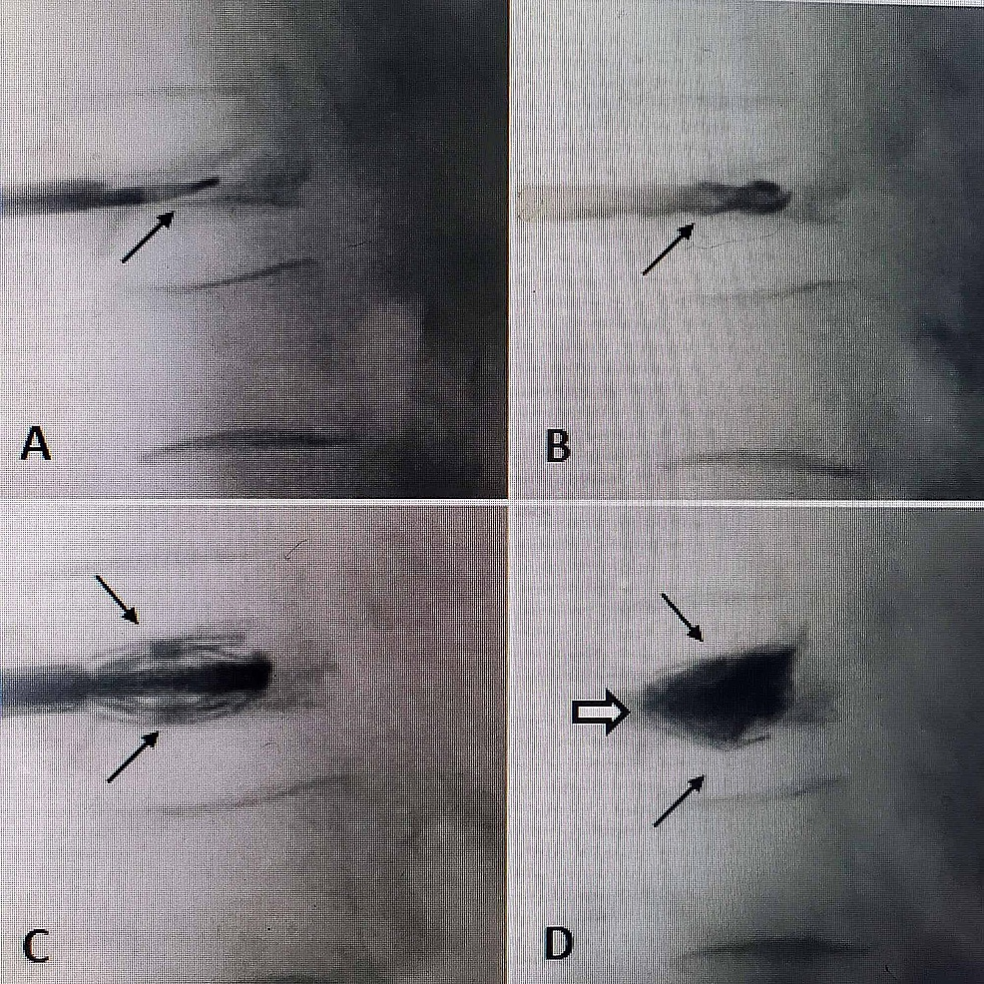
Sequential intra-operative films showing the vertebra plana with a dissecting curette passed directly along the vertebral cleft, followed by the 4.2 mm reamer, expanded SpineJack®, and cement. A: The access cannula and expandable curette (solid black arrow) passing directly into the center of the collapsed vertebra and cleft. B: The 4.2 mm reamer (solid black arrow), which is same size as the closed implant, is positioned from posterior-lateral through the pedicle to the anterior rim of the collapsed vertebra. C: The SpineJack® implants (solid black arrows) have been placed bilaterally and partially expanded, paused to allow gradual endplate expansion. D: The fully expanded implants (solid black arrows) filled with PMMA cement (black outlined white arrow) have led to 60% expansion of both the middle and especially anterior part of the VP collapse. PMMA, Polymethyl-methacrylate; VP, vertebra plana.

The most important anatomic/surgical considerations in VP are the following: (1) the pedicle is usually intact, rarely collapses, as well as supports the posterior wall of the vertebra. This anatomic/pathologic observation provides a clear pathway into the collapsed vertebra. Anatomically the superior and medial walls of the pedicle are thickest and aid in maintaining the posterior vertebral wall [6]. Initial reports of vertebroplasty for VP had a significant percentage of cases that approached the vertebrae translateral through the vertebral body and not the pedicle, but it is definitely possible to access the collapsed vertebrae always through the vertebral pedicle [5,6,8]. Technically, as long as the cannulas are guided parallel and in line with the VP as well as not at an angle so as not to pierce or pass through the endplate, it is feasible to direct the entire closed implant assembly within the collapsed vertebra. The additional use of a malleable curette as shown helps delineate the path and also the vertebral cleft. The placement of these implants angled from posterior-lateral at the pedicle to medial anteriorly also adds bilateral permanent structural support to the middle and anterior parts of the already weakened vertebrae [8]. During the procedure, gradual alternating expansion of the SJ devices is important to allow movement of the endplates and deployment of the “wings” along the more lateral walls of the vertebra in line with the pedicles under the superior endplate and over the inferior endplate from anterior to posterior spreading the load and opening the more biconcave central and anterior collapse. This gradual expansion also allows for a ligamentotaxis and aids the gradual retraction of any posteriorly displaced fragments [4,12,15]. When planning the surgical approach to VP fractures, by using all the radiologic studies, it is possible to maximize the degree of fracture re-expansion and angle correction (Figure 2).

## Conclusions

In this review of six patients with VP osteoporotic compression fractures that had different size SpineJack implants, it is clear that it is technically feasible to place these implants, even the larger 4.2 and 5.0 mm size, without any additional technical issues or risks compared to less severely collapsed vertebral fractures. The implants restore some degree of vertebral height even in severely collapsed vertebrae plana. However, it is key to understand that the height improvement is strictly based on the size of the implant, which varies from 12.5 to 20 mm with fully expanded implants between 4.2 mm and 5.8 mm; therefore, in making preoperative evaluation and measurement for sizing, the implant consideration should be given to placing the largest size appropriate to the pedicle in each case. Of the four patients that had posterior displaced fragments of the superior vertebral endplate, two of the six that had pre-procedure leg pain resolved their radicular pain. Follow-up CT scans in two patients showed reduction of the size of the fragment combined with partial correction of kyphosis related to the phenomenon of ligamentotaxis that occurs as the SJ expands and tightens the PLL. Although this series was small, all cases were performed under local anesthesia with mild sedation at an ambulatory surgical center or outpatient hospital setting. There was no intradiscal or epidural leak of cement, and to date with six- to 24-month follow-up there were no adjacent level fractures. These cases and our overall experience with VP fractures in general indicate that it is possible to place cannulas/balloons and specifically the larger SpineJack expandable implants bilaterally without any technical issues. The ability to re-expand a VP fracture and correct the kyphosis combined with the advantage of a permanent internal support of the osteoporotic-weakened VP fracture with cement fixation leads to more lasting fracture deformity correction and hopefully lowers the incidence of adjacent level fractures as seen in previous non-VP studies.
